# Cellular and Circuitry Bases of Autism: Lessons Learned from the Temporospatial Manipulation of Autism Genes in the Brain

**DOI:** 10.1007/s12264-017-0112-7

**Published:** 2017-03-07

**Authors:** Samuel W. Hulbert, Yong-hui Jiang

**Affiliations:** 1 Department of Neurobiology, School of Medicine, Duke University, Durham, NC 27710 USA; 2 Department of Pediatrics, School of Medicine, Duke University, Durham, NC 27710 USA; 3 Program in Genetics and Genomics, School of Medicine, Duke University, Durham, NC 27710 USA

**Keywords:** Autism, Mouse models, Behavior, Cre-*loxP*, Cerebellum, Critical period

## Abstract

Transgenic mice carrying mutations that cause Autism Spectrum Disorders (ASDs) continue to be valuable for determining the molecular underpinnings of the disorders. Recently, researchers have taken advantage of such models combined with Cre-*loxP* and similar systems to manipulate gene expression over space and time. Thus, a clearer picture is starting to emerge of the cell types, circuits, brain regions, and developmental time periods underlying ASDs. ASD-causing mutations have been restricted to or rescued specifically in excitatory or inhibitory neurons, different neurotransmitter systems, and cells specific to the forebrain or cerebellum. In addition, mutations have been induced or corrected in adult mice, providing some evidence for the plasticity and reversibility of core ASD symptoms. The limited availability of Cre lines that are highly specific to certain cell types or time periods provides a challenge to determining the cellular and circuitry bases of autism, but other technological advances may eventually overcome this obstacle.

## Introduction

The neurobiological basis of Autism Spectrum Disorders (ASDs) has been a growing area of research during the past few decades. While substantial progress has been made to uncover the molecular underpinnings of the disorders, the cell types and circuits underlying autistic behaviors remain largely unknown [1]. This knowledge, however, is critical to developing targeted therapy. Neuroimaging in human patients has provided correlates between the function of certain brain regions and behavior [2, 3]. Such studies are limited by the phenotypic heterogeneity that is characteristic of ASDs, the individuals available for study, the low resolution of imaging techniques, and the inability to manipulate molecules and circuits in the human brain. For these reasons, it is technically challenging to establish causality between circuits and behavior in studies involving humans. However, animal models allow researchers to determine causation because the genetic background and environmental factors can be controlled. Furthermore, the use of laboratory rodents allows for more invasive studies that would not be feasible or ethical in humans.

Transgenic mice with ASD-causing mutations that are present in the germline have provided clues to the molecular underpinnings of the disorders, and some degree of overlap is seen across multiple mouse models [4]. However, germline, or conventional, mutant mice do not provide enough evidence to causally link a particular brain region or circuit to ASD-like behaviors. More recently, several groups have taken advantage of tools that allow for the manipulation of genes across space and time in animal models to begin dissecting the cell types, brain regions, and associated circuits contributing to ASD phenotypes. In this review, we have focused on studies that utilize conditional gene-expression technology combined with mouse models of ASD that have high construct validity (i.e. the molecular consequences of the mutations mimic those observed in humans) and report phenotypes that have strong face validity (i.e. appear to mimic the behavioral features of the disorders) for the core symptoms of ASDs, impaired social communication and repetitive behaviors.

## Assays for ASD-Related Behaviors

Currently, ASDs are diagnosed purely on the assessment of behavioral features [5]. Therefore, behavioral analysis is an important part of the strategy to model human mutations and understand the cellular and circuitry bases of the disorders. To help achieve this goal, a number of assays that test behaviors resembling the core symptoms of ASDs have been developed for laboratory mice [6, 7]. The behaviors mentioned in this review are briefly described here and are summarized in Table 1.

**Table 1:** Summary of behavioral assays.

Assay	Resemblance to core feature of ASDs	Quantitative measurements
Three chamber test	Social communication deficits	Phase 1: “Sociability” - preference for social stimulus (stranger mouse) over an inanimate object Phase 2: “Social novelty” - preference for an unfamiliar mouse over the stranger mouse used in the sociability phase
Habituation/dishabituation	Social communication deficits	Time spent interacting with the same mouse during the trial period over several days and time spent with a novel mouse on the last trial
Partition test	Social communication deficits	Time spent near a barrier that separates the test mouse from a stranger mouse
Direct social interaction tests	Social communication deficits	Duration, type (e.g. aggressive), and amount of social interactions
Ultrasonic vocalizations	Social communication deficits	Number, duration, and complexity of vocalizations
Nest-building	Social communication deficits	Amount of nesting material used, size of nest, or numerical scoring system
Self-grooming	Repetitive behavior	Time spent grooming or number of grooming bouts
Jumping	Repetitive behavior	Time spent jumping or number of jumping bouts
Digging	Repetitive behavior	Time spent digging or number of digging bouts
Hole board test	Repetitive behavior	Number of total hole-pokes or number of consecutive (2+) pokes in the same hole
Marble-burying test	Repetitive behavior	Number of marbles buried

### Tests to Assess Social Communication

The three chamber test consists of two phases. The first phase has a caged stranger mouse in one of the chambers, a novel object in the opposing chamber, and a neutral central chamber [8]. The second phase has the caged mouse from the first phase in one chamber, a neutral central chamber, and a novel caged mouse in the opposing chamber. The first phase is considered a test for “sociability” where wild-type mice tend to spend more time in the chamber with the caged stranger mouse than in either other chamber. The second phase is a test for “preference for social novelty” or “social preference,” where most wild-type mice spend more time with the novel stranger mouse than with the familiar stranger mouse or in the neutral chamber.

The habituation/dishabituation test involves introducing the test mouse to the same contained stimulus mouse over several days and then introducing a novel contained stimulus mouse [9]. Wild-type mice tend to spend less time engaging with the familiar mouse over time, but have increased interactions with the novel mouse. This is a measure of social recognition.

In the partition test, the test mouse and a stimulus mouse are in an arena on opposite sides of a clear acrylic barrier with holes [10, 11]. The amount of time the test mouse spends near the partition is a measure of social behavior.

In direct social interaction tests, the test mouse and stimulus mouse are allowed to freely interact. The type, duration, and amount of contacts are scored [12]. One variant of this test is resident-intruder, where the stranger mouse is introduced into the test mouse’s home cage [13].

Ultrasonic vocalizations (USVs) can be recorded in pups (~postnatal days 3–12) and in adults [14]. For pups, this simply involves briefly separating the pups from their mother. For adults, the most common test involves exposing male mice to females in estrous or their urine. The number, duration, and complexity of USVs can be compared.

Nest-building is considered to be another measure of social behavior. The test mouse is given a small amount of cotton or other material to build a nest in its home cage. The amount of material used, the size of the nest, or a scoring system can be used to compare experimental groups [15].

### Tests to Assess Repetitive Behavior

Spontaneous repetitive behaviors include excessive self-grooming, jumping, or digging [16]. The time spent engaging in each behavior or number of bouts can be compared.

The hole board test also measures repetitive behavior. Mice are placed on an apparatus with circular holes arranged in a grid [17]. Mice that display repetitive behavior have more nose-pokes into the holes, or explore the same hole repeatedly.

Finally, in the marble burying test, the test mouse is placed in a clean cage with marbles positioned on top of bedding [18]. Mice with repetitive behavior bury more marbles in the bedding than their wild-type littermates.

## Tools to Manipulate Gene Expression

The Cre-*loxP* system is the approach taken most often to manipulate gene expression in a cell-type-specific or time-dependent manner [19]. For cell-type-specific manipulations, this method typically involves breeding mice that contain the *Cre recombinase* transgene under the control of a specific promoter with mice that have the target gene flanked by *loxP* sequences (floxed) [20]. Deletions, and rarely duplications, of the target DNA occur when the *loxP* sequences are oriented in the same direction in either the *cis* or *trans* configuration, whereas inversions occur if the *loxP* sequences are oriented in opposite directions. For more precisely-timed manipulations, the Cre recombinase protein is fused to a mutated estrogen receptor, so that tamoxifen treatment causes translocation into the nucleus and subsequent site-specific recombination [21]. Other, but less common, methods for inducible gene expression include the Tet-Off (tTA) and Tet-On (rtTA) systems, where doxycycline treatment represses and activates transcription, respectively [22].

While extraordinarily useful for temporospatial manipulation of genes in mice, the Cre-*loxP* system is not without its limitations. The major limiting factor is the availability of Cre lines that meet the needs of an experiment, in terms of timing and spatial distribution of Cre expression. The specificity and efficiency of recombination relies on the promoter, which may be expressed at various levels in off-target tissues or cell-types. One way to circumvent this problem is to deliver a lentivirus or adeno-associated virus construct containing Cre to mice with the target gene floxed [23]. While this method may limit the expression of Cre to a particular brain region, it is more difficult to control the expression of Cre in different cell types within a particular region. Another potential problem is that Cre expression itself can induce a phenotype by causing recombination at cryptic *loxP* sites in the mouse genome [24], or by affecting the expression of surrounding genes, depending on the insertion site of the transgene. Therefore it is important that proper controls are implemented to compare the phenotypes.

New advances in gene-editing technologies may eventually overcome the limitations of the Cre-*loxP* and similar systems. For example, it has been demonstrated that CRISPR/Cas9 can be used to edit the genome of adult mice and rescue disease phenotypes [25]. Moreover, methods are being developed to increase the cell-type specificity of CRISPR/Cas9-mediated mutations [26]. However, off-target mutations using this system remain a concern and must be assessed individually [27]. Thus, some refinement of this newer technology is necessary before using it to answer questions about the neuroanatomical bases of ASDs.

## The Roles of Excitatory and Inhibitory Neurons in ASDs

The two major classes of neurons in the brain are excitatory neurons, those which depolarize the neurons they project onto and thus make them more likely to produce action potentials, and inhibitory neurons, those which hyperpolarize their outputs and make them less likely to produce action potentials. It has been proposed that a common mechanism underlying ASDs is an imbalance between inhibitory and excitatory synaptic transmission in particular circuits. This may reflect a relative increase of function in excitatory neurons compared to that of inhibitory neurons [28], or *vice versa*. Although support for this hypothesis is currently somewhat limited, studies have begun to address it.

One important question is whether disrupting the function of either major class of neurons is sufficient to produce phenotypes related to ASDs and, if so, which types of neurons within these major classes contribute to the phenotypes. Another important question is whether different ASD-causing mutations affect the same neuronal types and circuits, or have shared circuit-level mechanisms. This may translate into whether patients with different mutations require individualized therapy in the clinic. Fortunately, the advent of conditional gene expression technologies and the production of multiple mouse models of ASDs with high construct validity have made it possible to start exploring these questions.

## Manipulations in Large Groups of Excitatory (Glutamatergic) Neurons

Most of the published work on conditional gene expression for ASDs involves manipulation of the mouse version of the genes that are mutated in syndromic autism, such as Rett syndrome (*MECP2*), tuberous sclerosis complex (*TSC1* or *TSC2*), *PTEN*-related disorders, and Angelman syndrome (*UBE3A*). One group found that restricting the deletion of *Mecp2* (*loxP/y*) to excitatory neurons in the forebrain postnatally, starting around postnatal day 21 (P21), with Tg(Camk2a-cre)93Kln (CamKII-cre93; EMMA 01137) results in mice that display reduced sociability in a test similar to the first phase of the three chamber test and reduced preference for the novel mouse in the habituation-dishabituation test [29]. However, another group used Emx1^tm1(cre)Krj^ (Emx1-cre; JAX 005628) to delete *Mecp2* (*loxP/y*) in forebrain excitatory neurons and glia embryonically, starting around embryonic day 9.5 (E9.5), and found no significant changes in either sociability or preference for social novelty in the three chamber test [30]. Neither study reported any repetitive behaviors. Deletion of *Mecp2* (*loxP/y*) selectively in glutamatergic neurons, the most abundant neurotransmitter class of excitatory neurons, in embryos with Slc17a6^tm2(cre)Lowl^ (Vglut2-cre; JAX 028863) does not impact social behavior in the partition test or nose-pokes on the hole board [31]. However, selectively expressing *Mecp2* (*loxP-stop/y*) in glutamatergic neurons with the same Cre line rescues a hypersocial phenotype on the partition test and repetitive nose-pokes on the hole board that occur in the global knockout, which suggests that restoring function to glutamatergic neurons may have network effects that subsequently increase the function of other excitatory neurotransmitters or inhibitory neurons as well [31].

On the other hand, mice that carry a deletion of another ASD candidate gene*, Mef2c* (*loxP/loxP*) with Emx1-cre spend less time interacting with the social stimulus in the first phase of the three chamber test, emit fewer USVs as pups and adults, have lower nest-building scores, and display increased repetitive jumping and fine motor movements [32]. Similarly, deletion of *Cc2d1a* (*loxP/loxP*) with Tg(CamK2a-cre)T29-1Stl (CamKIIα-cre T29-1; JAX 005359) results in mice that display reduced sociability in the first phase of the three chamber test, reduced social approach in a direct social interaction test, reduced numbers of adult USVs, and increased repetitive self-grooming (but no changes in marble-burying) [33]. Mice with *Tsc1* (*loxP/loxP*) deleted with CamKIIα-cre T29-1 also display reduced sociability in the three chamber test and increased marble-burying behavior [34].

Together, these findings indicate that disrupting the function of excitatory neurons can be sufficient to produce ASD-like behaviors, but conflicting findings between models indicate that manipulating one gene may not be generalizable to all ASDs. Therefore, studies using conditional gene expression of other ASD candidate genes are necessary to obtain a more complete understanding.

## Inhibitory (GABAergic) Neurons

Although there is some evidence that the dysfunction of excitatory neurons contributes to ASD-like phenotypes, there is accumulating evidence for a role of inhibitory neurons as well. Using the line Tg(Slc32a1-cre)2.1Hzo (Viaat-cre; JAX 017535) to delete *Mecp2* (*loxP/y*) specifically in GABAergic cells in embryos results in mice that show nearly the full spectrum of phenotypes observed in the conventional knockout, including repetitive forelimb stereotypies, increased grooming, sequential head-pokes on the hole board task, decreased nest-building, and increased sociability in the partition test and the three chamber test [35]. Accordingly, selectively restoring *Mecp2* (*loxP-stop/y* and *loxP-stop/+*) to GABAergic cells with Viaat-cre rescues the hypersocial phenotype in the partition test and nest-building deficits in both male and female mice (but repetitive behaviors were not reported) [36]. Moreover, deleting *Mecp2* (*loxP/y*) in a subset of GABAergic cells in the forebrain, embryonically, with Tg(Dlx6a-cre)1Mekk (Dlx5/6-cre; JAX 008199) recapitulates the nest-building, hole board, and hypersocial phenotypes, but not grooming, indicating that some features of ASD may involve other brain regions or cell types [35].

Two of the major subclasses of GABAergic neurons are parvalbumin-positive (PV+) and somatostatin-positive (SOM+) neurons [37]. Thus, one important question is whether either or both of these cell types contribute to ASD phenotypes. Mice that lack *Mecp2* (*loxP/y*) in PV+ neurons, embryonically, created by crossing the floxed mice with Pvalb^tm1(cre)Arbr^ (PV-cre; JAX 008069), display a hypersocial phenotype on the partition test, but show no differences on the hole board [38]. In contrast, mice that lack *Mecp2* (*loxP/y*) in SOM+ neurons, embryonically, created by crossing floxed mice with Sst^tm2.1(cre)Zjh^ (SOM-cre; JAX 013044), display repetitive nose-pokes on the hole board, but have no social phenotype [38]. A different study found no differences in sociability in mice lacking *Mecp2* in PV+ neurons, using the same PV-cre [39]. However, another study confirmed that dysfunctional PV+ neurons contribute to social phenotypes whereas SOM+ neurons do not by heterozygous deletion of the mouse version of the gene underlying Dravet syndrome, *Scn1a* (*loxP/+*), in either type of neuron using PV-cre and SOM-cre, respectively [40]. In this case, the mice lacking *Scn1a* in PV+ neurons show decreased sociability in the three chamber test [40].

It is likely, given that the conditional knockouts restricting *Mecp2* deletion to GABAergic neurons almost completely recapitulate all of the phenotypes observed in the conventional knockout, that the pathogenesis of Rett syndrome primarily involves dysfunctional inhibitory neural networks. Some behavioral deficits have also been reported in conditional *Mecp2* knockouts where the deletion is limited to excitatory neurons, but the decrease in social behavior is distinct from the global knockout, which shows increased social interest in the partition test. It is less clear at this time whether this is generalizable to other ASDs. Therefore, more research is needed that uses conditional gene expression technology and highly penetrant ASD-causing mutations.

## Other Neurotransmitter Systems

Other neurotransmitters can either be excitatory or inhibitory, depending on the action of the postsynaptic receptors that are expressed. Since the majority of neurons in the brain are either glutamatergic or GABAergic, less is known about the roles of other neurotransmitters in ASD models. However, some groups have started dissecting the roles of dopamine, serotonin, acetylcholine, and oxytocin.

Restricting the deletion of *Mecp2* (*loxP/y*) to serotonergic neurons, embryonically, with Tg(Fev-cre)1Esd (PET-1 cre; JAX 012712) results in mice that display a hypersocial phenotype on the partition test as well as increased aggression during the resident-intruder test [41]. On the other hand, selectively deleting *Mecp2* (*loxP/y*) in dopaminergic neurons, embryonically, with Th^tm1(cre)Te^ (TH-cre; EMMA 00254) does not influence social behavior in these assays [41]. The PET-1 cre knockout does not have repetitive grooming or marble-burying, but these behaviors were not assessed in the TH-cre knockout [41]. This study, along with others, suggests that dysfunctional serotonergic neurons may contribute to the core symptoms of ASDs, whereas dopaminergic neurons are implicated in comorbid motor functions [42–44]. More support for the role of serotonergic neurons in ASD behaviors comes from deleting *Tsc1* (*loxP/loxP*) selectively in serotonergic neurons, embryonically, with Tg(Slc6a4-cre)ET33Gsat (Slc6a4-cre; MMRRC 031028-UCD), which results in mice with decreased sociability in the first phase of the three chamber test and increased repetitive marble-burying behaviors [34].

Recently, the cholinergic system has also been implicated in the pathogenesis of ASD with conditional gene expression in mice. Selectively deleting *Mecp2* (*loxP/y*) in cholinergic neurons, embryonically, with Chat^tm2(cre)Lowl^ (Chat-cre; JAX 006410) produces mice that fail to show a preference for social novelty in the three chamber test, show decreased social investigation in a direct interaction test, and have impaired nest-building [45]. Interestingly, re-expression of *Mecp2* (*via* microinjection of an AAV) in the basal forebrain, but not in the caudate-putamen, of adult mice rescues the social deficit in the three chamber test [45]. Again, this phenotype is distinct from that in the global *Mecp2* knockout, but nonetheless provides insight into circuits underlying ASDs.

So far, the role of oxytocinergic neurons is less clear. Neither heterozygous (*loxP/+*) nor homozygous (*loxP/loxP*) deletion of *Pten* in oxytocinergic neurons, embryonically, with Oxt^tm1.1(cre)Dolsn^ (Oxt-cre; JAX 024234) results in deficits in the three chamber, habituation/dishabituation, or marble-burying tests [46]. This is somewhat puzzling, given that *Pten* haploinsufficient, oxytocin knockout, and oxytocin receptor knockout mice show some overlapping impairments on these assays [47–50]. Perhaps mutations in *PTEN* and other ASD-causing mutations have more of an effect on neurons expressing the oxytocin receptor, rather than on oxytocinergic neurons. More work needs to be done to clarify the role of oxytocin and other neurotransmitters in ASDs.

## The Role of the Cerebellum in ASDs

In recent years, the cerebellum has emerged as a brain region that may be implicated not only in motor impairments, but also in the core behaviors of ASDs, particularly because cerebellar Purkinje cells project to the thalamus and, for example, affect dopamine efflux in the prefrontal cortex [51]. Mice with heterozygous (*loxP/+*) or homozygous (*loxP/loxP*) deletion of *Tsc1* mostly restricted to Purkinje cells, postnatally, by means of Tg(Pcp2-cre)2Mpin (L7-cre; JAX 004146) fail to demonstrate preference for a social stimulus over a non-social stimulus or for novel social interactions over familiar ones in the three chamber test [52]. These mice also engage in elevated rates of self-grooming [52]. Similarly, mice with *Tsc2* homozygously deleted in Purkinje cells with L7-cre and heterozygously deleted in all other cell types (loxP/-) have impaired social interactions and increased rates of marble-burying [53]. Mice with *Pten* deleted (*loxP/loxP*) in Purkinje cells by L7-Cre display reduced sociability and engage in repetitive upright scrabbling, but have reduced self-grooming [54]. Interestingly, deleting *Pten* (*loxP/loxP*) primarily in cerebellar granule cells with Tg(Gfap-cre)1Sbk (Gfap-cre) results in mice that are hyposocial in the partition test and show reduced sociability in the three chamber test, but show reduced repetitive behaviors in the marble-burying and hole board tests, and have no changes in USVs as pups [55].

Most recently, two similar studies produced opposite conclusions regarding the cerebellum’s role in social behavior. Purkinje cell deletion of exon 7 of *Shank2* (*loxP/loxP*) with L7-Cre resulted in mice that failed to demonstrate a preference for sociability or for social novelty [56]. However, mice with a deletion of exons 6-7 of *Shank2* (*loxP/loxP*) with Tg(Pcp2-cre)3555Jdhu (Pcp2-cre; JAX 010536) were similar to controls on the three chamber task and had similar USVs, but had increased nose-pokes on the hole board [57]. Both groups of mice showed normal levels of self-grooming and marble-burying.

The divergent findings regarding social behavior between the two studies using *Shank2* conditional mutants have several possible explanations. One is that minor differences in the exonic deletions could disrupt the expression of different sets of Shank2 isoforms, but current knowledge regarding the structure of *Shank2* suggests this unlikely to be the case [58]. Another possibility is that the use of different Cre lines (JAX 004146 vs. JAX 010536) in the two studies may have some effect on behavior; although both Cre lines involve the same promoter, there may be differences in the spatial pattern and timing of Cre expression. The most likely explanation is that the two labs used different methods for determining behavioral phenotypes (for example, strains chosen for the stranger mice and habituation times). This last explanation is especially convincing because the wild-types in one study showed a preference for social novelty [56], whereas those in the other study did not [57], most likely indicating differences in behavioral methods.

Further investigation into the cerebellum’s contributions to ASD phenotypes is clearly warranted. Moreover, the contradictory findings from similar studies underscore the need for stricter standards for behavioral methods or more side-by-side comparisons between lines of mice within investigating laboratories to make the strongest conclusions. It is unclear at this time whether, for instance, *Tsc1, Tsc2, Pten,* and *Shank2* deletions in the cerebellum have different effects on self-grooming, or whether the discrepancies between the studies are due to varying methods. It is even less clear which cerebellum-related circuitry is responsible for the ASD-like behaviors and whether there is any translational value of these findings for human patients.

## Developmental Time Points Implicated through Inducible Mutations and Rescues

Clinically, ASDs are classified as a group of neurodevelopmental disorders. However, the developmental origin of ASDs has not been clearly defined. This knowledge is likely critical for effective clinical intervention. Conditional gene expression, through inducible mutations and rescues, allows researchers to determine whether ASDs are the result of disrupted development and/or ongoing neuronal dysfunction. Conditional-rescue mice also provide proof of principle for the potential of gene therapy. One attractive hypothesis for the origin of ASDs is that perturbations to synaptic development prevent patients from developing skills during a limited window of opportunity, or a critical period [59]. This would suggest that reversing the symptoms of ASD may be difficult, if not impossible. Some studies suggest that early intervention is necessary for preventing the onset of ASD-like phenotypes. However, multiple studies have also challenged the notion that ASDs represent an irreversible disruption of brain development.

Deleting *Mecp2* in adulthood (*loxP/y*) using the ubiquitously-expressed, inducible Cre line, Tg(CAG-cre/Esr1*)5Amc (CAGGS-CreER; JAX 004453) and tamoxifen administration, causes the impaired nest-building phenotype that is observed in germline knockouts [60]. Reactivating *Mecp2* in male (*loxP-stop/y*) and female (*loxP-stop/+*) model mice with the same Cre line and tamoxifen administration partially and completely rescue this nest-building impairment, respectively [61]. Neither of these studies reported any other ASD-related behaviors, nor did two other studies that reactivated *Mecp2* expression [62, 63]. However, reinstating *Ube3a* (*loxP-stop/p+*) using the same inducible Cre line fails to rescue impaired nest-building in these mice, even when tamoxifen is administered to newborn mice; only embryonic reinstatement of *Ube3a* successfully rescues this phenotype [64].

On the other hand, several ASD-related behaviors are rescued by repressing the expression of a dominant-negative form of *Nrxn1β* in excitatory neurons in adulthood using Tg(Camk2a-tTA)1Mmay (CaMKII-tTA; JAX 003010) with the administration of doxycycline, which reverses increased self-grooming and impaired sociability and preference for social novelty in the three chamber test [65]. Similarly, re-expressing normal *Shank3* isoforms by reverting an inverted portion of the gene after administering tamoxifen to adult mice (*loxP/loxP*; CAGGS-CreER) rescues increased grooming, impaired sociability in the first phase of the three chamber test, and reduced preference for social novelty in the second phase of this test [66].

While in general these studies provide some promising evidence for the reversibility of the core symptoms of ASDs, research using other models and additional ASD-relevant behaviors is necessary to make more definitive conclusions. An interesting and important question is whether the findings from mouse models can be translated to humans and if the reversibility of symptoms holds true.

## Concluding Remarks and Future Directions

It is clear that, at least in some cases, disrupting the function of either excitatory or inhibitory neurons is sufficient to cause the core behaviors of ASD. Likewise, manipulations of either forebrain or cerebellar neurons can induce ASD-like phenotypes in mice. The study of specific regions outside of the cerebellum is one particularly important direction for future study. For example, several studies have implicated the striatum in the pathophysiology of ASDs by showing region-specific molecular and electrophysiological alterations [67, 68]. Moreover, suppression of Shank3 expression using shRNA targeted to the ventral tegmental area can induce social deficits [69]. However, specific circuits have not yet been causally linked to ASD-relevant behaviors using cell-type specific Cre lines.

The findings reviewed here (summarized in Table 2) support the value of conditional gene-expression technology to delineate the cells and circuits underlying ASDs. However, because these studies are mostly conducted in syndromic ASD models, it remains to be seen whether these findings and conclusions are unique to these specific ASD-causing mutations. Since there are inconsistencies between studies that manipulate the same gene, it may be premature to generalize the limited available evidence. Human genetics studies have supported the role of several hundreds of genes in ASDs, so one challenge is to find out whether there are shared circuit-level mechanisms among ASDs caused by different mutations or etiologies. Caution must be taken when interpreting the results from studies that utilize conditional gene expression. Too often the efficiency and specificity of the Cre lines are overestimated. Moreover, disruption of one set of cells may have downstream or compensatory effects on other cell types in the same brain region, or even in different brain regions due to axonal projections. Hopefully, the development of more precise technology for manipulating cell types and circuits will facilitate a more complete understanding of the neural underpinnings of ASDs.

**Table 2:** Summary of transgenic mouse lines used for the temporospatial manipulation of ASD genes in the mouse brain.

Cre line (or “Tet-Off” line)	Commercial availability	Cells targeted	Timing of gene expression manipulation	Social communication phenotypes	Repetitive behavior phenotypes	References
CamKII-cre93	EMMA 01137	Excitatory forebrain neurons	Postnatal (~P21)	*Mecp2 (loxP/y)* ↓ Sociability* ↓ Social novelty*** NR USVs NR nest-building	*Mecp2 (loxP/y)* NR grooming NR hole board NR marble-burying	Cre line primary: [70] *Mecp2* study: [29]
CamKIIα-cre T29-1	JAX 005359	Excitatory forebrain neurons	Postnatal (~P21)	*Cc2d1a* (*loxP/loxP*) ↓ Sociability*, **** NR social novelty ↓ Adult USVs NR nest-building *Tsc1* (*loxP/loxP*) ↓ Sociability* NR social novelty NR USVs NR nest-building	*Cc2d1a* (*loxP/loxP*) ↑ Grooming NR hole board ↔ Marble-burying *Tsc1* (*loxP/loxP*) NR grooming NR hole board ↑ Marble-burying	Cre line primary: [20] *Cc2d1a* study: [33] *Tsc1* study: [34]
Emx1-cre	JAX 005628	Excitatory forebrain neurons and glia	Embryonic	*Mecp2 (loxP/y)* ↔ Sociability* ↔ Social novelty* NR USVs NR nest-building *Mef2c (loxP/loxP)* ↓ Sociability* NR social novelty ↓ Pup, adult USVs ↓ Nest-building	*Mecp2 (loxP/y)* NR grooming NR hole board NR marble-burying *Mef2c (loxP/loxP)* NR grooming NR hole board NR marble-burying	Cre line primary: [71] *Mecp2* study: [30] *Mef2c* study: [32]
Vglut2-cre	JAX 028863	All glutamatergic neurons	Embryonic	*Mecp2 (loxP/y)* ↔ Sociability** NR social novelty NR USVs NR nest-building *Mecp2 (loxP-stop/y)* ↑ Sociability** resc. NR social novelty NR USVs NR nest-building	*Mecp2 (loxP/y)* NR grooming ↔ Hole board NR marble-burying *Mecp2 (loxP-stop/y)* NR grooming ↑ Hole board resc. NR marble-burying	Cre line primary: [72] *Mecp2* study: [31]
Viaat-cre	JAX 017535	All GABAergic neurons	Embryonic	*Mecp2 (loxP/y)* ↑ Sociability*, ** NR social novelty NR USVs ↓ Nest-building *Mecp2 (loxP-stop/y)* ↑ Sociability** resc. NR social novelty NR USVs ↓ Nest-building resc.	*Mecp2 (loxP/y)* ↑ Grooming ↑ Hole board NR marble-burying *Mecp2 (loxP-stop/y)* NR grooming NR hole board NR marble-burying	Cre line primary: [35] *Mecp2* deletion study: [35] *Mecp2* rescue study: [36]
Dlx5/6-cre	JAX 008199	Subset of GABAergic neurons in forebrain	Embryonic	*Mecp2 (loxP/y)* ↑ Sociability*, ** NR social novelty NR USVs ↓ Nest-building	*Mecp2 (loxP/y)* ↔ Grooming ↑ Hole board NR marble-burying	Cre line primary: [73] *Mecp2* study: [35]
PV-cre	JAX 008069	PV+ GABAergic neurons	Embryonic	*Mecp2 (loxP/y)* ↑ Sociability** ↔ Sociability* NR social novelty NR USVs NR nest-building *Scn1a (loxP/+)* ↓ Sociability* NR social novelty NR USVs NR nest-building	*Mecp2 (loxP/y)* NR grooming ↔ Hole board NR marble-burying *Scn1a (loxP/+)* NR grooming NR hole board NR marble-burying	Cre line primary: [74] *Mecp2* studies: [38, 39] *Scn1a* study: [40]
SOM-cre	JAX 013044	SOM+ GABAergic neurons	Embryonic	*Mecp2 (loxP/y)* ↔ Sociability** NR social novelty NR USVs NR nest-building *Scn1a (loxP/+)* ↔ Sociability* NR social novelty NR USVs NR nest-building	*Mecp2 (loxP/y)* NR grooming ↑ Hole board NR marble-burying *Scn1a (loxP/+)* NR grooming NR hole board NR marble-burying	Cre line primary: [75] *Mecp2* study: [38] *Scn1a* study: [40]
PET-1 cre	JAX 012712	All serotonergic neurons	Embryonic	*Mecp2 (loxP/y)* ↑ Sociability** ↑ Aggression**** NR social novelty NR USVs NR nest-building	*Mecp2 (loxP/y)* ↔ Grooming NR hole board ↔ Marble-burying	Cre line primary: [76] *Mecp2* study: [41]
Slc6a4-cre	MMRRC 031028-UCD	All serotonergic neurons	Embryonic	*Tsc1 (loxP/loxP)* ↓ Sociability* NR social novelty NR USVs NR nest-building	*Tsc1* (*loxP/loxP*) NR grooming NR hole board ↑ Marble-burying	Cre line primary: [77] *Tsc1* study: [34]
TH-cre	EMMA 00254	All dopaminergic neurons	Embryonic	*Mecp2 (loxP/y)* ↔ Sociability** ↔ Aggression**** NR social novelty NR USVs NR nest-building	*Mecp2 (loxP/y)* NR grooming NR hole board NR marble-burying	Cre line primary: [78] *Mecp2* study: [41]
Chat-cre	JAX 006410	All cholinergic neurons	Embryonic	*Mecp2 (loxP/y)* ↓ Sociability**** ↓ Social novelty* NR USVs ↓ Nest-building	*Mecp2 (loxP/y)* NR grooming NR hole board NR marble-burying	Cre line primary: [79] *Mecp2* study: [45]
Oxt-cre	JAX 024234	All oxytocinergic neurons	Embryonic	*Pten* (*loxP/+*) and Pten (*loxP/loxP)* ↔ Sociability*, *** ↔ Social novelty*, *** NR USVs NR nest-building	*Pten* (*loxP/+*) and Pten (*loxP/loxP)* NR grooming NR hole board ↔ Marble-burying	Cre line primary: [80] *Pten* study: [46]
L7-cre	JAX 004146	Cerebellar Purkinje cells	Early postnatal	*Tsc1* (*loxP/+*) and *Tsc1* (*loxP/loxP*) ↓ Sociability* ↓ Social novelty* NR USVs NR nest-building *Tsc2* (*loxP/-)* ↓ Sociability* ↓ Social novelty* NR USVs NR nest-building *Pten (loxP/loxP)* ↓ Sociability* NR social novelty NR USVs NR nest-building *Shank2e6-7 (loxP/loxP)* ↓ Sociability* ↓ Social novelty* NR USVs NR nest-building	*Tsc1* (*loxP/+*) and *Tsc1* (*loxP/loxP*) ↑ Grooming NR hole board NR marble-burying *Tsc2* (*loxP/-)* NR grooming NR hole board ↑ Marble-burying *Pten (loxP/loxP)* ↓ Grooming NR hole board NR marble-burying *Shank2e7 (loxP/loxP)* ↔ Grooming NR hole board ↔ Marble-burying	Cre line primary: [81] *Tsc1* study: [52] *Tsc2* study: [53] *Pten* study: [54] *Shank2* study: [56]
Pcp2-cre	JAX 010536	Cerebellar Purkinje cells	Early postnatal	*Shank2e7 (loxP/loxP)* ↔ Sociability* ↔ Social novelty* ↔ Adult USVs NR nest-building	*Shank2e6-7 (loxP/loxP)* ↔ Grooming ↑ Hole board ↔ Marble-burying	Cre line primary: [82] *Shank2* study: [57]
Gfap-cre	N/A	Mostly cerebellar granule cells, few hippocampal neurons	Unclear	*Pten (loxP/loxP)* ↓ Sociability*, ** NR social novelty ↔ Pup USVs NR nest-building	*Pten (loxP/loxP)* NR grooming ↓ Hole board ↓ Marble-burying	Cre line primary: [83] *Pten* study: [55]
CAGGS-CreER	JAX 004453	Ubiquitous	Concurrent with tamoxifen administration (P60+ for all examples)	*Mecp2* (*loxP/y*) NR sociability NR social novelty NR USVs ↓ Nest-building *Mecp2* (*loxP-stop/y*) and *Mecp2* (*loxP-stop/+*) NR sociability NR social novelty NR USVs ↓ Nest-building resc. *Ube3a (loxP-stop/p+)* NR sociability NR social novelty NR USVs ↓ Nest-building no resc. *Shank3 (loxP/loxP)* inverted ↓ Sociability* resc. ↓ Social novelty* resc. NR USVs NR nest-building	*Mecp2* (*loxP/y*) NR grooming NR hole board NR marble-burying *Mecp2* (*loxP-stop/y*) and *Mecp2* (*loxP-stop/+*) NR grooming NR hole board NR marble-burying *Ube3a (loxP-stop/p+)* NR grooming NR hole board ↓ Marble-burying no resc. *Shank3 (loxP/loxP)* inverted ↑ Grooming resc. NR hole board NR marble-burying	Cre line primary: [84] *Mecp2* deletion study: [60] *Mecp2* rescue study: [61] *Ube3a* study: [64] *Shank3* study: [66]
CaMKII-tTA	JAX 003010	Excitatory forebrain neurons	Concurrent with doxycycline administration (P60+ for this example)	*TRE-HAβnrx1ΔC* ↓ Sociability* resc. ↓ Social novelty* resc. NR USVs NR nest-building	*TRE-HAβnrx1ΔC* ↑ Grooming resc. NR hole board NR marble-burying	Tet-Off line primary: [85] *Nrxn1β* study: [65]

*NR* behavioral test not reported, *resc.* abnormal phenotype was rescued with genetic manipulation, *no resc.* genetic manipulation failed to rescue abnormal phenotype, *JAX* The Jackson Laboratory, *EMMA* The European Mouse Mutant Archive, *MMRRC* Mutant Mouse Resource & Research Centers.

* In the three chamber test; ** in the partition test; *** in the habituation/dishabituation test; **** in a direct social interaction test.

↑ Mutant mice have significantly elevated amounts of this behavior when compared to controls.

↓ Mutant mice have significantly decreased amounts of this behavior when compared to controls.

↔ There were no significant differences between mutants and their controls on this behavioral test.
